# Assessment of Orbital Compartment Pressure: A Comprehensive Review

**DOI:** 10.3390/diagnostics12061481

**Published:** 2022-06-16

**Authors:** Tim J. Enz, Markus Tschopp

**Affiliations:** 1 Department of Ophthalmology, University of Basel, CH-4031 Basel, Switzerland; 2 Lenzburg Eye Clinic, CH-5600 Lenzburg, Switzerland; 3 Department of Ophthalmology, Cantonal Hospital Aarau, CH-5001 Aarau, Switzerland; markus.tschopp@windowslive.com; 4 Department of Ophthalmology, Inselspital, University of Bern, CH-3010 Bern, Switzerland

**Keywords:** orbital compartment pressure, minimally invasive measurement, orbital compartment syndrome, thyroid eye disease

## Abstract

The orbit is a closed compartment defined by the orbital bones and the orbital septum. Some diseases of the orbit and the optic nerve are associated with an increased orbital compartment pressure (OCP), e.g., retrobulbar hemorrhage or thyroid eye disease. Our aim was to review the literature on the different approaches to assess OCP. Historically, an assessment of the tissue resistance provoked by the retropulsion of the eye bulb was the method of choice for estimating OCP, either by digital palpation or with specifically designed devices. We found a total of 20 articles reporting direct OCP measurement in animals, cadavers and humans. In nine studies, OCP was directly measured in humans, of which five used a minimally invasive approach. Two groups used experimental/custom devices, whilst the others applied commercially available devices commonly used for monitoring the compartment syndromes of the limbs. None of the nine articles on direct OCP measurements in humans reported complications. Today, OCP is mainly estimated using clinical findings considered surrogates, e.g., elevated intraocular pressure or proptosis. These diagnostic markers appear to reliably indicate elevated OCP. However, particularly minimally invasive approaches show promises for direct OCP measurements. In the future, more sophisticated, specifically designed equipment might allow for even better and safer measurements and hence facilitate the diagnosis and monitoring of orbital diseases.

## 1. Introduction

The orbit is a closed compartment defined by the orbital bones and the orbital septum, containing a number of structures essential for the visual process. As such, the orbit is subject to a certain compartment pressure. Some diseases of the orbit are associated with an increased orbital compartment pressure (OCP), a key clinical finding leading to compression and the subsequent damage of the intraorbital structure, e.g., retrobulbar hemorrhage or thyroid eye disease [1,2,3]. Furthermore, it has been postulated that the OCP is a determining factor of intraocular pressure and optic nerve subarachnoid space pressure, and hence of the lamina cribrosa pressure gradient [4,5]. Thus, the OCP may be important in the pathogenesis of glaucoma or in estimating intracranial pressure based on optic disc signs. Accurate measurements of the pressure dynamics within the orbital compartment may hence be helpful for enhancing our understanding of glaucoma and other optic neuropathies possibly associated with altered OCP.

Historically, an assessment of the tissue resistance provoked by the retropulsion of the eye bulb was the method of choice for estimating OCP, either by digital palpation or with specifically designed devices [6,7]. Today, with widely experimental methods [8,9,10,11,12,13,14,15,16,17,18,19,20,21,22,23,24,25,26,27,28], the direct measurement of OCP is considered technically feasible yet impractical in a clinical setting due to limited availability, difficult arrangement, and invasiveness. Thus, in suspected orbital compartment syndrome, for example, OCP is generally not measured directly. Rather, it is estimated based on clinical findings that are considered surrogates, e.g., proptosis or the intraocular pressure [1,9,29,30].

Since only few attempts have been made to directly measure the OCP, there is a paucity of evidence regarding physiologic and pathologic OCP values. Consequently, no criteria for a threshold OCP for decompression exist either. To the best of our knowledge, the sparse literature on this topic has never been reviewed. The purpose of this study is to exploratively review the literature on indirect assessments of OCP as well as to systematically review the literature on technical modalities and results of direct OCP measurements, and hence to provide a comprehensive overview of the different approaches to OCP assessment.

## 2. Materials and Methods

**Figure 1:**
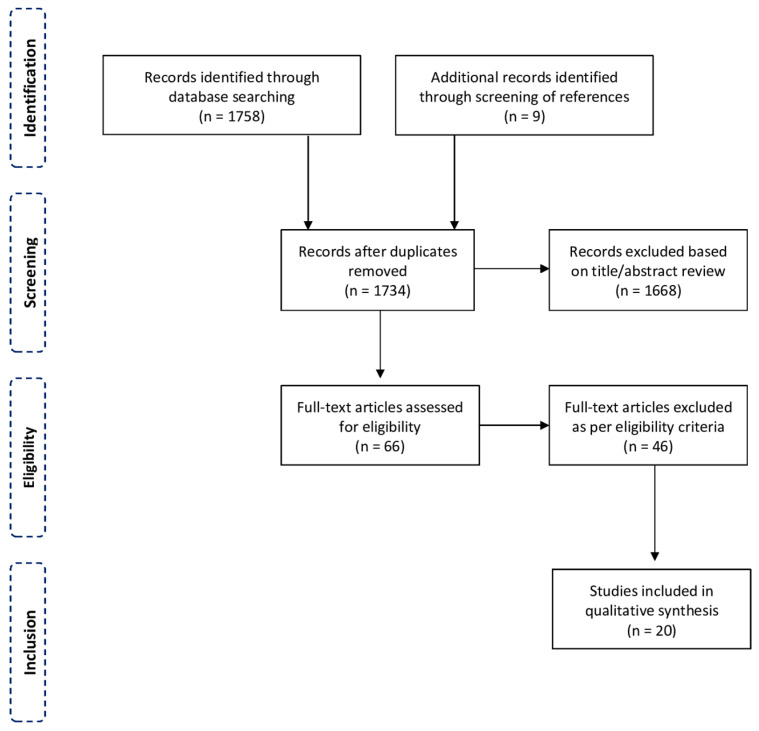
Flowchart showing the methodology of the systematic review.

An exploratory literature search on the indirect assessment of the OCP was conducted and the results are summarized narratively from a historical perspective (see *Results*). The PubMed database was systematically searched for articles related to the direct measurement of OCP. The following search algorithm was used: ((retrobulbar) OR (orbital) OR (intraorbital)) AND (pressure) AND ((measurement) OR (manometry) OR (assessment) OR (evaluation) OR (monitoring) OR (transducer)). Additionally, references were screened for potential articles of interest. The latest search was performed on 20 October 2021. All retrieved publications were reviewed. To be eligible, the articles had to (a) present original data on the direct measurement of OCP, (b) report at least one outcome of interest, (c) be written English, German or French, and (d) be published in peer-reviewed journals. Reports on adult and pediatric patients were eligible, as well as anatomical and animal studies. Full texts were reviewed for studies with questionable eligibility. The search methodology is displayed in Figure 1.

## 3. Results

### 3.1. Indirect Orbital Compartment Pressure Assessments: Historical Perspectives

Historically, an assessment of the tissue resistance provoked by the retropulsion of the eye bulb was considered the method of choice for estimating the pressure within the orbital compartment. In 1868, von Graefe recommended digital retropulsion to evaluate the ease with which the globe can be retropulsed in patients with suspected thyroid eye disease [6]. In 1910, Langenhan introduced the first device to measure and quantify orbital tissue resistance during ocular retropulsion [31], followed by Gutmann [32], Plegge [33], and Georg [34], who assessed the weight necessary to retropulse the globe in patients in a supine position. However, only Copper’s instrument described in 1948 was used more extensively to indirectly measure the OCP [7]. With Copper’s orbitotonometer, the OCP was measured by putting weights in 100 g increments up to 400 g on a rod resting on the orbital rims of a patient lying in a supine position, exerting force on the eye bulb that had previously been anesthetized and covered with a contact lens. Baseline exophthalmometer values, the globe position relative to the orbital rim at various applied forces, and the graph generated when plotting the ocular position against the amount of force applied were the parameters assessed by Copper’s method. In this setting, the amount of force was pre-given while the extent of retropulsion was the independent variable. In the following years, a number of reports on the clinical application of Copper’s orbitotonometer for monitoring thyroid eye disease and orbital tumors were published by various investigators [35,36,37,38]. In 1968, Elsby described a device for measuring orbital compliance, which appeared to be, however, very similar to Copper’s orbitotonometer [39]. In 1975, Doege described an orbitotonometer with electronic recordings of the obtained data. Although this led to many advantages, the working principle remained the same [40].

A different approach to quantify OCP was described only in 1984 by McGowan et al., who developed a technique involving swim goggles. Fluid was added to the space between the goggles and the eye alongside continuous electronic measurements of the pressure inside this space [41,42]. With the pressure increase plotted against the volume added to the space over time, conclusions on the OCP were drawn. Frueh and associates revived the principle of using a contact lens for transmitting force onto the eye bulb and measuring OCP. However, in their setting, the patients were seated in an upright position, eliminating gravity-associated effects. The device retropulsed the eye bulb at a certain distance and measured the forces generated using pressure transducers, making the extent of retropulsion the pre-given variable and the amount of applied force the independent variable. Thus, the method allowed for much more accurate OCP measurements and a better comparison between different patients and eyes. Using this technique, Frueh et al. indirectly assessed OCP in healthy subjects and patients with orbital diseases. However, given the complicated arrangement and the lack of convenience for the patients, the technique could not establish itself as routine clinical practice [43].

### 3.2. Direct Orbital Compartment Pressure Measurements: Systematic Review

Our initial systematic search on direct OCP measurements identified 1758 articles. After the removal of duplications and title and abstract screening, a total of 66 articles remained. Next, full texts were thoroughly checked for eligibility and references were screened for additional publications of interest, resulting in a total of 20 articles from 1985 to 2021 for the final inclusion [8,9,10,11,12,13,14,15,16,17,18,19,20,21,22,23,24,25,26,27,28]. The reviewed papers and main data are shown in Table S1. Four articles reported studies using animals (New Zealand white rabbits, goats, Macaca fascicularis monkeys, and Yorkshire piglets) [13,14,15,21] and seven studies used human cadaver heads for experiments [11,17,18,20,24,26,28]. Only nine articles reported direct OCP measurements in living humans [8,9,10,12,16,19,22,23,27]. Of these, four articles reported pressure measurements during major orbital surgery [8,16,19,27]. Five articles reported minimally invasive measurements in an outpatient setting, two of them by the same group of investigators [9,10,12,22,23].

In the reviewed studies, a total of 70 animals (124 animal orbits) were investigated. These studies were conducted to assess the interactions between OCP and orbital physiology and pathophysiology in different simulated orbital conditions, yet were not primarily to explore the feasibility of the measurement procedure itself. The devices used for measuring the OCP were either experimental (involving catheters, spherical expanders and pressure transducers) [13,14,21] or, in one case, a commercially available single-use device designed for measuring compartment pressures in limbs [15]. Nevertheless, their findings allowed for a number of highly interesting conclusions, e.g., regarding the significance of the OCP for bony orbital growth stimulation [14,21] or the amount of energy absorbed by the intraorbital soft tissue following orbital trauma (see Table S1).

In the seven anatomical studies, a total of 35 human cadaver heads (64 orbits) were investigated. The cadaver heads were mostly used to simulate orbital compartment syndrome (by orbital hemorrhage or space-occupying lesions). Typically, the authors aimed to assess the efficacy of different decompression techniques (canthotomy, cantholysis, septolysis, etc.). Again, either custom devices using pressure catheters and pressure transducers or commercially available tools for monitoring compartment pressure in limbs were used. The authors frequently concluded that certain decompression techniques are viable for lowering OCP in orbital compartment syndrome and that some procedures are superior to others (see Table S1) [11,18,26,28].

A total of 167 patients (219 orbits) were enrolled in the nine studies on living humans. Mostly, these studies were conducted either to test for feasibility of measuring devices or to assess OCP dynamics in orbital compartment syndrome or thyroid eye disease before or following orbital decompression [8,9,10,12,16,19,22,23,27].

Four groups of researchers investigated OCP changes following major orbital decompression surgeries [13,14,15,21]. Kratky and colleagues were among the first to directly measure the OCP in 1990. They assembled a custom pressure measuring device consisting of a slit catheter and a pressure transducer to measure changes in OCP in patients with different orbital diseases, as well as in patients with healthy orbits during major orbital surgeries (enucleation, exenteration, or orbital decompression). Their study aimed to establish a normal range of OCP (3–6 mmHg). Since normal subcutaneous tissue pressure levels are sub-atmospheric, the authors concluded that their findings support the concept that the orbit indeed functions as an enclosed compartment. Furthermore, their study showed that the OCP can be elevated in thyroid eye disease (7–15 mmHg) [16]. Similarly, in 1996, Otto and associates aimed to investigate OCP dynamics in patients with thyroid eye disease during surgical decompression. To that purpose, they used a self-assembled custom device consisting of a micro-pressure transducer placed into the orbit and attached to a research amplifier and a chart recorder [19]. Based on their findings, the authors concluded that thyroid eye disease with optic neuropathy is associated with much higher pressures than without optic neuropathy, and that a pressure decrease occurs immediately following decompression surgery. In 2010, Berthout et al. used a Codman© Pressure Monitor commonly used for measuring the intracranial pressure, and they investigated changes in OCP in patients with thyroid eye disease during orbital decompression procedures. Their findings confirm that OCP is significantly increased in severe thyroid eye disease and that it can be reduced or normalized with surgical decompression of the orbit [8]. Zhou et al. also attached a retrobulbar catheter to a pressure transducer and a graphical and numerical recorder. Using this custom device, they assessed the OCP in patients with orbital wall fractures during surgical repair (see Table S1) [27].

Only four groups of investigators (five articles) attempted to measure the OCP minimally invasively [9,10,12,22,23]. Egbert and colleagues, as well as Riemann et al., assembled custom devices to that purpose [10,22,23]. Riemann et al. used a 23-gauge blunt-tipped retrobulbar needle to which a pressure transducer kit commonly used for measuring hemodynamic parameters was attached. This transducer was connected to a patient monitoring system which yielded alphanumeric and graphic representations of the pressure. Using this custom device, the investigators assessed OCP dynamics during intraorbital anesthetic injection for ocular surgery. Their studies aimed to determine a physiologic range of OCP in patients with healthy orbits (6.3 ± 1.7 mmHg (mean ± Standard Deviation)) as well as with thyroid eye disease (9.7 ± 4.8 mmHg (mean ± Standard Deviation)). In addition, the authors concluded that intraorbital anesthetic injections consistently cause changes in OCP and that directly assessing OCP dynamics in vivo may prove useful both as an adjunct in the clinical evaluation of patients with disorders resulting in orbital compartment syndrome, as well as in assessing the risk of retrobulbar injection in orbits at greater risk of complications from this procedure [22,23]. Egbert’s device consisted of a specially designed cannula which was made with side ports for piezometric tappings. At these side ports, pressure values were measured and the OCP was calculated from these values during corticosteroid injections into the orbital capillary hemangiomas. Their findings suggest that intralesional pressure may rise tremendously during injections (range 18.65–842.2 mmHg) and may routinely exceed systemic arterial pressures. Since a sufficient volume of corticosteroid injected at a high injection pressure would account for the embolization of corticosteroid particles into the ocular circulation from the retrograde arterial flow, the authors concluded that the volume of corticosteroid to be injected should be limited and indirect ophthalmoscopy should be performed on all patients receiving injections of long-acting corticosteroids into the orbit and periorbital soft tissue [10]. On the other hand, Czyz and Strand, as well as Enz and associates, used commercially available devices commonly used for minimally invasively monitoring of the compartment pressure of the limbs. Both groups used a Compass Compartment Monitor© device for measuring OCP in real and simulated orbital compartment syndrome [9,12]. Czyz and Strand assessed a single patient with clinical signs of orbital compartment syndrome and recorded an OCP value of 14 mmHg [9]. Enz et al. used patients scheduled for ocular surgery under peribulbar anesthesia as a human in vivo model of orbital congestion. They assessed OCP changes before and during intraorbital anesthetic injections and reported a mean baseline pressure value of 2.5 ± 1.5 mmHg (mean ± standard deviation) and an increase to 12.8 ± 9.2 mmHg (mean ± standard deviation) following injection [12]. Both groups came to the conclusion that the direct measurement of OCP using this device appears feasible and useful in diagnosing and monitoring patients with suspected orbital compartment syndrome.

None of the nine articles on direct OCP measurements in humans reported complications associated with the measuring process.

**Figure 2:**
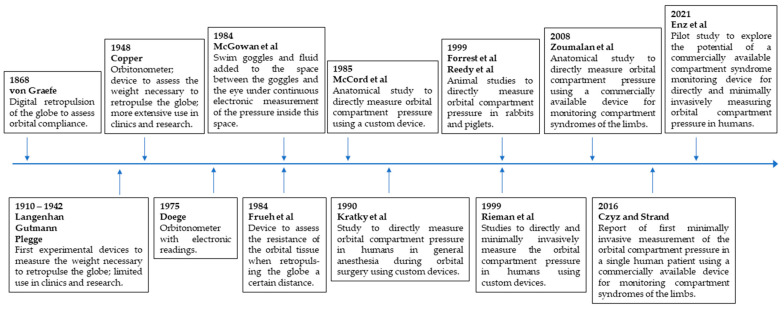
Timeline of the development of the different methods to measure orbital compartment pressure.

The chronological sequence of the development of the different methods to measure orbital compartment syndrome is displayed in Figure 2.

### 3.3. Clinical Findings Considering Surrogates for Elevated Orbital Compartment Pressure

Today, orbital compartment pressure is usually not measured directly. Instead, in clinical situations, it is mainly estimated using clinical findings that are considered surrogates for elevated OCP. Decisions against or in favor of possible therapeutic interventions are usually based only on these clinical surrogates, occasionally supported by findings of imaging procedures (CT, MRI) showing evidence of retrobulbar fluid accumulation, pronounced proptosis, extraocular muscle enlargement, or optic nerve compression [1,9,29,30,44].

In suspected orbital compartment syndrome, raised intraocular pressure (IOP) is recognized as one of the most important of those clinical signs, besides visual disturbances, tight orbits, proptosis, ocular motility restrictions, choroidal folds, or signs of optic nerve compression [1,9,12,28,45]. The underlying rationale is that the orbit is a closed compartment and, thus, space-occupying intraorbital lesions affect both the orbital compartment and intraocular pressure similarly [28,46]. Indeed, Oester et al. and Zoumalan et al. reported correlating changes in OCP and IOP in their cadaver-based model of orbital compartment syndrome [18,28]. The findings of Zoumalan’s group suggested a steady increase in both OCP and IOP starting from 3 mL intraorbital volume increments [28]. Zhou et al. and Enz et al. assessed the relationship between OCP and IOP changes in living humans and found similar correlations [12,27]. Furthermore, Enz et al., as well as Stanley et al., also found a correlation between elevations in OCP and the extent of proptosis, concluding that proptosis can be considered a valid surrogate as well [12,24].

## 4. Discussion

### 4.1. Clinical Signs of Elevated Orbital Compartment Pressure as Diagnostic Markers

While digital palpation of the globe and the periocular tissue continues to be commonly performed as an indirect approach for assessing tight orbits, the devices described above for quantifying the resistance provoked by the retropulsion of the eye bulb did not endure time, given their complicated arrangement and the lack of convenience for the patients, and can thus be considered obsolete.

Assessment of the clinical signs that are considered markers for elevated OCP (elevated IOP, proptosis, motility restrictions, etc.) is relatively easy, fast, and cheap, and hence widely available. A number of studies aimed to assess the correlation of these clinical findings with OCP and thus to test their validity as surrogates. The groups of Oester and Zoumalan both reported correlating changes in OCP and IOP in their cadaver-based orbital compartment syndrome model before and after surgical decompression [18,28]. However, it remains uncertain whether these results are applicable to living humans, in which autonomic pressure regulation mechanisms are likely to respond to an initial rise in IOP. Enz et al. measured OCP dynamics in living humans during intraorbital anesthetic injection and found similar correlations, but they did not assess the course of the pressure values in the minutes and hours following the injection [12]. In fact, the increase in IOP observed by Khan et al., one minute after an intraorbital fluid injection, was only transient. Within the following ten minutes, the IOP reached sub-baseline levels [47]. Moreover, according to anecdotal reports, severe orbital compartment syndrome with distinct proptosis, ocular motility restriction and visual loss can manifest while IOP is only slightly elevated or even within normal limits [9,48]. Thus, the relationship between changes in OCP and IOP is yet to be fully clarified. In a retrospective chart review by Erickson et al., it was found that proptosis and many other traditionally emphasized clinical markers failed to identify the patients in need of surgical intervention [49]. In summary, published evidence might allow for the preliminary conclusion that elevated IOP can be seen as a valid indicator for elevated OCP, yet normal IOP may not be suitable for excluding elevated OCP (limited sensitivity). The same may apply for proptosis as an indicator for elevated OCP, yet evidence in this respect is even scarcer.

In case of suspected orbital compartment syndrome with potential vision loss, surgical decompression must not be delayed and the indication for surgical intervention should be made at a low threshold [1,2,30,45,50,51]. However, studies have shown that emergency physicians, who are often the first to provide care in cases of facial trauma, are particularly reluctant to perform lateral canthotomy, as they feel insecure when interpreting clinical ophthalmological signs and believe they need more training to diagnose orbital compartment syndrome correctly [52,53,54]. Thus, the need for additional diagnostic capabilities and guidance is often expressed by non-ophthalmologists [49,52,53]. A practical and safe modality for directly measuring OCP would therefore be of great value.

### 4.2. Direct Orbital Compartment Pressure Measurements and Paucity of Data

Despite the paramount clinical importance of OCP in many respects, only few attempts have been made to directly measure it. Our search identified only nine articles reporting direct OCP measurements in humans. Consequently, there is a paucity of data on ranges of pressure values in health and disease, and no established threshold pressure values for surgical intervention have been determined yet either. So far, no equipment has been designed and manufactured specifically for direct OCP measurements. Hence, the mentioned measurements were performed using either experimental, self-assembled equipment, or devices that are commonly used for monitoring compartment syndromes of the limbs or intracranial parenchymatous pressure. Interestingly, none of the studies on direct OCP measurements in humans reviewed in our study reported safety issues associated with the measuring process. Yet, all of them claimed to have gained valuable findings and insights into OCP dynamics. Consequently, the authors of the 20 reviewed studies typically concluded that direct OCP measurement may be feasible and provide useful clinical and diagnostic information.

Regarding the measured pressure values, comparisons and generalizations are difficult given the pronounced heterogeneity concerning the experimental settings, real or simulated health conditions, and measuring devices. Although the data on OCP values is limited, published evidence may allow for approximation of a typical range of pressure values in health (2–6 mmHg) and disease (thyroid eye disease: up to 40 mmHg and higher; orbital compartment syndrome: up to 100 mmhg and higher). The existing literature further allows for the conclusion that OCP can be effectively released or even normalized by many common decompression techniques (canthotomy, cantholysis, septolysis, etc.) (see Table S1). These pressure dynamics are apparently directly measurable, and thereby gained data appear feasible for monitoring the progression of orbital diseases or the outcome of treatments.

Notably, however, the orbit is divided into different sub-compartments by muscles and septa. Hemorrhages or interstitial fluid may not evenly spread across the different sub-compartments and, thus, OCP may not be the same in all parts of the orbit, possibly limiting the diagnostic value of direct pressure measurements. On the other hand, the OCP has been suggested as a determining factor of IOP and optic nerve subarachnoid space pressure, and hence of the lamina cribrosa pressure gradient. As such, OCP may play a role in the pathogenesis of glaucoma and other optic neuropathies [4,5]. Theoretically, precise measurements of OCP could enhance our understanding of the pathogenesis of these diseases and possibly even serve as a biomarker.

### 4.3. Future Developments

Assessment of the indirect clinical markers of elevated OCP is relatively easy, fast, inexpensive, and hence widely available. Furthermore, these surrogates appear to relatively reliably indicate elevated OCP in orbital compartment syndrome. Thus, assessing these clinical findings will continue to be part of the management of orbital diseases. In many cases, these indirect clinical findings allow for diagnosis and therapeutic decision making sufficiently reliably without the need for further testing. In cases of suspected orbital compartment syndrome with potential vision loss, the indication for surgical intervention should be made at a low threshold. However, the results of our literature review indicate that all published experimental attempts to directly measure OCP using custom devices or devices commonly used for other purposes were considered successful by the investigators, and provided useful clinical and diagnostic information. Particularly minimally invasive approaches appear promising for directly measuring OCP. Thus, in the future, more sophisticated, custom-made equipment might allow for even safer and more precise direct, minimally invasive OCP measurements and facilitate the diagnosis of orbital compartment syndrome or thyroid eye disease. Furthermore, such measurement modalities may prove helpful in the research and assessment of other optic neuropathies possibly associated with altered orbital compartment pressure.

**Figure 3:**
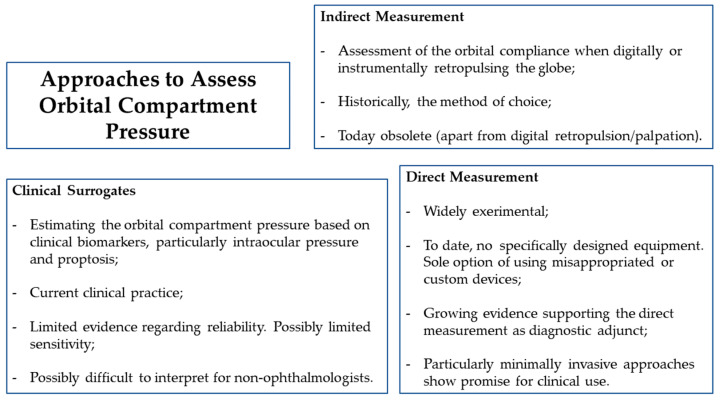
Overview of the different approaches to assess orbital compartment pressure and the corresponding main conclusions.

An overview of the different approaches to assess orbital compartment pressure is shown in Figure 3.

## 5. Conclusions

In conclusion, to date, the diagnosis and monitoring of elevated OCP is based on clinical signs considered as surrogates, particularly, elevated IOP and proptosis. These established indirect clinical diagnostic markers appear to be reliable indicators for elevated OCP. However, growing evidence supports direct OCP measurement as a diagnostic adjunct. Particularly minimally invasive approaches show promise for routine use. To date, no device/equipment has been specially designed for this purpose; hence, clinicians are left with the sole option of using either inappropriate or experimental equipment. In the future, more sophisticated, specifically designed equipment might allow for even better and safer direct, minimally invasive OCP measurements, and hence facilitate the diagnosis and monitoring of orbital diseases and optic neuropathies possibly associated with altered orbital compartment pressure.

## Data Availability

Publicly available datasets were analyzed in this study.

## Supplementary Materials

The following supporting information can be downloaded at: https://www.mdpi.com/article/10.3390/diagnostics12061481/s1, Table S1. Systematic review of articles related to direct orbital compartment pressure measurement.

## References

[1] Lima V., Burt B., Leibovitch I., Prabhakaran V., Goldberg R.A., Selva D. (2009). Orbital compartment syndrome: The ophthalmic surgical emergency. Surv. Ophthalmol..

[2] McCallum E., Keren S., Lapira M., Norris J.H. (2019). Orbital Compartment Syndrome: An Update With Review Of The Literature. Clin. Ophthalmol..

[3] Saeed P., Tavakoli Rad S., Bisschop P.H.L.T. (2018). Dysthyroid Optic Neuropathy. Ophthalmic Plast. Reconstr. Surg..

[4] Berdahl J.P., Yu D.Y., Morgan W.H. (2012). The translaminar pressure gradient in sustained zero gravity, idiopathic intracranial hypertension, and glaucoma. Med. Hypotheses.

[5] Morgan W.H. (2013). Central venous pulsations: New findings, clinical importance and relation to cerebrospinal fluid pressure. J. Glaucoma.

[6] Von Graefe A. (1868). Zusatze uber intraocularen tumoren. Arch. Ophthalmol..

[7] Copper A.C. (1948). An Introduction to Clinical Orbitonometry.

[8] Berthout A., Vignal C., Jacomet P.V., Galatoire O., Morax S. (2010). Intraorbital pressure measured before, during, and after surgical decompression in Graves’ orbitopathy. J. Fr. Ophtalmol..

[9] Czyz C.N., Strand A.T. (2016). Minimally invasive in vivo orbital pressure measurement. Clin. Exp. Ophthalmol..

[10] Egbert J.E., Paul S., Engel W.K., Summers C.G. (2001). High injection pressure during intralesional injection of corticosteroids into capillary hemangiomas. Arch. Ophthalmol..

[11] Elpers J., Areephanthu C., Timoney P.J., Nunery W.R., Lee H.B.H., Fu R. (2021). Efficacy of vertical lid split versus lateral canthotomy and cantholysis in the management of orbital compartment syndrome. Orbit.

[12] Enz T.J., Papazoglou A., Tappeiner C., Menke M.N., Benitez B.K., Tschopp M. (2021). Minimally invasive measurement of orbital compartment pressure and implications for orbital compartment syndrome: A pilot study. Graefes Arch. Clin. Exp. Ophthalmol..

[13] Forrest C.R., Khairallah E., Kuzon W.M. (1999). Intraocular and intraorbital compartment pressure changes following orbital bone grafting: A clinical and laboratory study. Plast. Reconstr. Surg..

[14] Gilliland G.D., Gilliland G., Fincher T., Harrington J., Gilliland J.M. (2005). Assessment of biomechanics of orbital fracture: A study in goats and implications for oculoplastic surgery in humans. Am. J. Ophthalmol..

[15] Johnson D., Winterborn A., Kratky V. (2016). Efficacy of Intravenous Mannitol in the Management of Orbital Compartment Syndrome: A Nonhuman Primate Model. Ophthalmic Plast. Reconstr. Surg..

[16] Kratky V., Hurwitz J.J., Avram D.R. (1990). Orbital compartment syndrome. Direct measurement of orbital tissue pressure: 1. Technique. Can. J. Ophthalmol..

[17] McCord C.D., Putnam J.R., Ugland D.N. (1985). Pressure-volume orbital measurement comparing decompression approaches. Ophthalmic Plast. Reconstr. Surg..

[18] Oester A.E., Fowler B.T., Fleming J.C. (2012). Inferior orbital septum release compared with lateral canthotomy and cantholysis in the management of orbital compartment syndrome. Ophthalmic Plast. Reconstr. Surg..

[19] Otto A.J., Koornneef L., Mourits M.P., Deen-van Leeuwen L. (1996). Retrobulbar pressures measured during surgical decompression of the orbit. Br. J. Ophthalmol..

[20] Ramesh S., Bokman C., Mustak H., Lo C., Goldberg R., Rootman D. (2018). Medial Buttressing in Orbital Blowout Fractures. Ophthalmic Plast. Reconstr. Surg..

[21] Reedy B.K., Pan F., Kim W.S., Bartlett S.P. (1999). The direct effect of intraorbital pressure on orbital growth in the anophthalmic piglet. Plast. Reconstr. Surg..

[22] Riemann C.D., Foster J.A., Kosmorsky G.S. (1999). Direct orbital manometry in healthy patients. Ophthalmic Plast. Reconstr. Surg..

[23] Riemann C.D., Foster J.A., Kosmorsky G.S. (1999). Direct orbital manometry in patients with thyroid-associated orbitopathy. Ophthalmology.

[24] Stanley R.J., McCaffrey T.V., Offord K.P., DeSanto L.W. (1989). Space-occupying orbital lesions: Can critical increases in intraorbital pressure be predicted clinically?. Laryngoscope.

[25] Moradi A., Sepah Y.J., Ibrahim M.A., Sophie R., Moazez C., Bittencourt M.G., Annam R.E., Hanout M., Liu H., Ferraz D. (2014). Association of retinal vessel calibre and visual outcome in eyes with diabetic macular oedema treated with ranibizumab. Eye.

[26] Strand A.T., Czyz C.N., Gibson A. (2017). Canthal cutdown for emergent treatment of orbital compartment syndrome. Orbit.

[27] Zhou H., Fan X., Xiao C. (2007). Direct orbital manometry in normal and fractured orbits of Chinese patients. J. Oral Maxillofac. Surg..

[28] Zoumalan C.I., Bullock J.D., Warwar R.E., Fuller B., McCulley T.J. (2008). Evaluation of intraocular and orbital pressure in the management of orbital hemorrhage: An experimental model. Arch. Ophthalmol..

[29] Dolman P.J. (2021). Dysthyroid optic neuropathy: Evaluation and management. J. Endocrinol. Investig..

[30] Ballard S.R., Enzenauer R.W., O’Donnell T., Fleming J.C., Risk G., Waite A.N. (2009). Emergency lateral canthotomy and cantholysis: A simple procedure to preserve vision from sight threatening orbital hemorrhage. J. Spec. Oper. Med..

[31] Langenhan F. (1910). Instrumentelle Messung der Zurückdrängbarkeit der Augenapfel in die Augenhöhle. Z. Augenheilkd.

[32] Gutmann A. (1927). Piezonmeter, zur Diagnose retrobulbärer Orbitageschwülste. Z. Augenheilkd.

[33] Plegge H. (1931). Eine neue Methode zur Messung der Zurückdrängbarkeit des Bulbus in der Orbita (Piezometrie). Klin. Monatsbl. Augenheilkd.

[34] Georg F. (1942). Ein neues Piezometer. Arch. Ophthalmol..

[35] Means J.H., Stanbury J.B. (1950). Clinical orbitonometry. Am. J. Med. Sci..

[36] Kearns T.P., Henderson J.W., Haines S.F. (1953). Clinical orbitonometry in Graves’s disease. Am. J. Ophthalmol..

[37] Grossmann E.E., Bruns T.A. (1954). Clinical use of orbitonometry. Am. J. Ophthalmol..

[38] Dyer J.A., Henderson J.W. (1958). Orbitonometry in unilateral exophthalmos. Am. J. Ophthalmol..

[39] Elsby J.M. (1968). Orbitonometer. Br. J. Ophthalmol..

[40] Doege E. (1975). Orbitopiezography and its clinical significance (author’s transl). Klin. Monbl. Augenheilkd.

[41] McGowan H.D., Hurwitz J.J., Gentles W. (1984). Orbitotonography, the dynamic assessment of orbital tension: 1. Results in subjects without known orbital disease. Can. J. Ophthalmol..

[42] Hurwitz J.J., McGowan H.D., Gentles W., Weise R.A., Victor W. (1988). Orbitotonography, the dynamic assessment of orbital tension: 2. Results in patients with orbital disease. Can. J. Ophthalmol..

[43] Frueh B.R. (1984). Graves’ eye disease: Orbital compliance and other physical measurements. Trans. Am. Ophthalmol. Soc..

[44] Oester A.E., Sahu P., Fowler B., Fleming J.C. (2012). Radiographic predictors of visual outcome in orbital compartment syndrome. Ophthalmic Plast. Reconstr. Surg..

[45] Voss J.O., Hartwig S., Doll C., Hoffmeister B., Raguse J.D., Adolphs N. (2016). The “tight orbit”: Incidence and management of the orbital compartment syndrome. J. Craniomaxillofac. Surg..

[46] Nassr M.A., Morris C.L., Netland P.A., Karcioglu Z.A. (2009). Intraocular pressure change in orbital disease. Surv. Ophthalmol..

[47] Khan S.A., Alam M., Aftab A.M., Iqbal M. (2014). Comparison of the efficacy of subtenon with peribulbar local anesthesia without hyaluronidase in patients undergoing cataract surgery. J. Coll. Physicians. Surg. Pak..

[48] Leshner M., Gibbons R., Costantino T. (2018). Adult Male with Traumatic Eye Pain and Swelling. Clin. Pract. Cases Emerg. Med..

[49] Erickson B.P., Garcia G.A. (2020). Evidence-based algorithm for the management of acute traumatic retrobulbar haemorrhage. Br. J. Oral Maxillofac. Surg..

[50] Federico C., Mario I., Edoardo P., Luigi Branca V., Fausto C., Nicola de A., Andrea B., Andrew W.K., Massimo S., Paola F. (2020). Timing of surgical intervention for compartment syndrome in different body region: Systematic review of the literature. World J. Emerg. Surg..

[51] Murali S., Davis C., McCrea M.J., Plewa M.C. (2021). Orbital compartment syndrome: Pearls and pitfalls for the emergency physician. J. Am. Coll. Emerg. Physicians Open.

[52] Hislop W.S., Dutton G.N., Douglas P.S. (1996). Treatment of retrobulbar haemorrhage in accident and emergency departments. Br. J. Oral Maxillofac. Surg..

[53] Dixon J.L., Beams O.K., Levine B.J., Papas M.A., Passarello B.A. (2020). Visual outcomes after traumatic retrobulbar hemorrhage are not related to time or intraocular pressure. Am. J. Emerg. Med..

[54] Edmunds M.R., Haridas A.S., Morris D.S., Jamalapuram K. (2019). Management of acute retrobulbar haemorrhage: A survey of non-ophthalmic emergency department physicians. Emerg. Med. J. EMJ.

