# Supplementary material for: Assessment of Orbital Compartment Pressure: A Comprehensive Review
Source: Diagnostics (Basel). 2022 Jun 16;12(6):1481. doi: 10.3390/diagnostics12061481 (PMC9221953; doi:10.3390/diagnostics12061481)
Supplement: Supplementary file 1 [file diagnostics-12-01481-s001.zip › diagnostics-1718150-supplementary.pdf]

Table S1. Systematic review of articles related to direct orbital compartment pressure measurement.

Authors	Year	Title	# of Subjects/ Orbits	Human/Cadaver/ Animal/Artificial Orbits	Condition	Purpose	Measurement Technique	Measured values of orbital compartment pressure	Compli-cations	Conclusions by the authors	Implications for practice	Implications for research
**Berthout A et al**	2010	[Intraorbital pressure measured before, during, and after surgical decompression in Graves' orbitopathy]	12 / 20	Human	Thyroid Eye Disease	To investigate the changes in orbital compartment pressure in thyroid eye disease and following orbital decompression.	A Codman® device for intracranial parenchymatous pressure measurement was inserted transcutaneally into the orbit using an inferotemporal approach under general anesthesia. The orbital compartment pressure was measured before decompression and after decompression of each orbital wall.	Before orbital decompression<br>- overall: 14,05 mmHg ± 9,19 (mean±SD)<br>- compressive optic neuropathy (n=5): 26.8 mmHg ± 7.85 (mean±SD)<br>- no compressive optic neuropathy: 9.8 mmHg ± 4.2 (mean±SD)<br><br>After orbital decompression<br>- overall: 4.3 mmHg ± 2.53 (mean±SD)<br>- compressive optic neuropathy: 6.4 mmHg ± 2.07 (mean±SD)<br>- no compressive optic neuropathy: 3.6 mmHg ± 2.32 (mean±SD)<br><br>Total decrease induced by the surgery: 9.75±7.55mmHg (mean±SD); significantly greater for the group with vs. without optic neuropathy (-20.4 mmHg versus -6.2 mmHg (P < 0.001)). The pressure reduction was greater after collapse of the first wall (floor) than after collapse of following walls for all groups (P < 0.001). The maximum pressure observed during the intervention (caused by the instruments): 78.3±23.47mmHg (mean±SD) without pupillary changes.	none	Intraorbital pressure is increased in Thyroid Eye Disase, participating in the appearance of compressive optic neuropathy. Orbital decompression provides a significant reduction in orbital compartment pressure pressure.	Intraorbital compartment pressure is increased in Thyroid Eye Disease and may be decreased adequately by surgical decompression. Orbital compartment pressure may be measured directly using the Codman® device.	Feasibility of the Codman® device for direct measurement of orbital compartment pressure in outpatient settings without general anesthesia needs to be further tested.
**Czyz CN and Strand AT**	2016	Minimally invasive in vivo orbital pressure measurement.	1 / 1	Human	Retrobulbar hemorrhage after gunshot trauma	To present a case of orbital compartment syndrome, in which orbital compartment pressure was measured minimally-invasively using a Compass Compartment Pressure Monitoring device.	A Compass Compartment Pressure Monitor (Mirador Biomedical, Inc, Seattle, WA, USA) was advanced transconjunctivally 15-20mm posterior into the orbit in an anatomically safe zone.	14mmHg	none	The device used in this study allows for direct measurement of orbital compartment pressure in vivo in an emergency setting and may enhance the diagnostic capabilities in suspected orbital compartment syndromes.	In suspected orbital compartment syndrome, direct monitoring of orbital compartment pressure is possible using the Compass device.	Further research is necessary to establish a threshold pressure value for indication of decompression in orbital compartment syndrome due to retrobulbar hemorrhage.
**Egbert J et al**	2001	High injection pressure during intralesional injection of corticosteroids into capillary hemangiomas	3 /3	Human	Orbital capillary hemangiomas, intralesional corticosteroid injections	To investigate the mechanism of complications following intralesional injections of corticosteroids in tumours of the head and neck.	The pressure produced during injection of corticosteroid was estimated using specially designed cannula (standard 21-gauge stainless steel tube, 3.82 cm long, with a nominal internal diameter of 0.05 cm, and a sharp beveled tip) attached to the syringe used for injecting the corticosteroids into the orbital lesions. The cannula was made with side ports for piezometric tappings at 2 sites along the length of the tube. The orbital compartment pressure, "P3", was calculated from pressures "P1"and "P2" measured at these 2 side ports.	Maximum pressures during injections 18.65-842.2mmHg (range).	None	Injection pressures exceeding the systemic arterial pressures routinely occur during intralesional injections of corticosteroids into intraorbital capillary hemangiomas. A sufficient volume of corticosteroid injected at high injection pressure would account for the embolization of corticosteroid particles into the ocular circulation from retrograde arterial flow.	Recommendation to limit the volume of corticosteroid and perform indirect ophthalmoscopy on all patients receiving injections of long-acting corticosteroids into the orbit and periorbital soft tissue.	The potential of this custom pressure measuring device for measuring orbital compartment pressure in settings other than intralesional injections in capillary hemangiomas needs to be further explored.
**Elpers J et al**	2021	Efficacy of vertical lid split versus lateral canthotomy	4 / 7	Cadaver	Simulated Orbital	To compare the efficacy of the vertical lid split to	A compartment pressure monitor (Stryker Intra-Compartment Pressure Monitor System 295) was inserted into	In simulated orbital compartment syndromes: 75-148mmHg (range).	None	Vertical lid split is as effective as lateral canthotomy and cantholysis	Different decompression techniques may be feasible	The potential of the Stryker compartment pressure monitor for

		and cantholysis in the management of orbital compartment syndrome			Compartment Syndrome	the standard lateral canthotomy and cantholysis for orbital compartment syndrome in the cadaveric model	the superonasal orbit to a depth of 2 cm, and the initial reading was recorded in mmHg from the digital readout	One minute after different decompression techniques: 20-48 mmHg (range).<br><br>16 minutes after different decompression techniques: 16-37mmHg.		in lowering orbital compartment pressure in orbital compartment syndrome and may be used alternatively in clinical settings.	in orbital compartment syndromes.	measuring orbital compartment pressure in vivo needs to be further explored.
**Enz TJ et al**	2021	Minimally invasive measurement of orbital compartment pressure and implications for orbital compartment syndrome: a pilot study	20 / 20	Human	Simulated Orbital Congestion	To explore the feasibility of minimally invasive needle manometry for direct measurement of orbital compartment pressure under reproducible conditions in an in vivo model of orbital congestion and to evaluate intraocular pressure and proptosis as indicators for elevated orbital compartment pressure.	Orbital compartment pressure was measured by needle manometry. A commercially available Compass Compartment Pressure Monitor [Mirador Biomedical, Inc., Seattle, USA] single-use manometer device was inserted between the syringe and a 32 mm 23G needle used for anesthetic injection.	Baseline: 2.5 ± 1.5 mmHg (mean±SD)<br><br>After intraorbital injection of 7 ml anesthetic: 12.8 ± 9.2 mmHg (mean±SD)	None	Needle manometry using the Compass device presents as a feasible, safe and easy-to-use method for measuring orbital compartment pressure directly and minimally invasively in a clinical setting in vivo.	Orbital compartment pressure may be directly measured in clinical settings of suspected elevated orbital compartment pressure using this technique.	The feasibility of this technique for measuring orbital compartment pressure in real emergency cases needs to be further tested
**Forrest CR et al**	1999	Intraocular and Intraorbital Compartment Pressure Changes following Orbital Bone Grafting: A Clinical and Laboratory Study	29 / 58	Animal (New Zealand White Rabbits)	Simulation of orbital bone grafting by sequential insertion of polyethylene sheeting into the orbit.	To test the null hypothesis that intraocular and intraorbital compartment pressure values remain constant despite orbital volume reduction with graft material for simulating intraorbital bone grafting for the reconstruction of acute traumatic defects or long-standing enophthalmos.	Wick catheters were inserted through the superior fornix into the periorbital soft tissues and were maintained in position with 3-0 silk sutures. Intraorbital pressure was measured continuously on a standard transducer for 60 minutes.	Before graft insertion:<br>- controls: 9.4±8.2mmHg (mean±SD)<br>- acute orbital trauma 8.1±5.6mmHg (mean±SD)<br>- chronic orbital trauma: 18.1±7.4mmHg (mean±SD)<br>- intact orbits: 6.8±3.1mmHg (mean±SD)<br><br>At stabilized end points:<br>- controls: 16.0±1.1mmHg (mean±SD)<br>- acute orbital trauma: 17.4±5.1 (mean±SD)<br>- chronic trauma: 21.0±3.4mmHg (mean±SD)<br>- intact orbits: 18.0±9.1mmHg (mean±SD)	None	Orbital bone grafting is not associated with significant changes in orbital compartment pressure in this animal-based model. The human orbit, however, due to anatomical differences may be more prone to developping orbital compartment syndromes.	Patients with scarred orbital tissue may be more prone to developping elevated orbital compartment pressures as a result of decreased compliance of the orbital tissue.	Further research is necessary to explore the potential of the Wick catheter for measuring orbital compartment pressure in humans in vivo. Further research is required to determine if orbital bone grafting in humans is associated with significant changes in orbital compartment pressure.
**Gilliland GD et al**	2005	Assessment of biomechanics of orbital fracture: a study in goats and implications for oculoplastic surgery in humans	21 / 42	Animal (Goats)	Orbital trauma and orbital fracture	To determine the biophysical characteristics of energy absorption of the various orbital structures and the amount of force/energy required to produce an orbital fracture in goats, and to interpret the results in terms of human clinical applicability.	A catheter attached to a pressure transducer (Transpac IV; Abbott Laboratories) was inserted into the intraconal space of the orbit (inserted with a 23-gauge butterfly needle) to determine the temporal response of the traumatic impulse. This transducer has a 3-dB response at 1.5 kHz. These pressure profiles were then recorded and digitized for analysis.	Baseline: 1-2mmHg (range)<br><br>During impact: >20mmHg	None	Through intraorbital and intraocular pressures, we are able to quantify the amount of energy absorbed by the orbital soft tissue (globe displacement) before bony fracture as 50 to 75 mJ.	This study is applicable in predicting the presence or absence of a fracture on the basis of the mechanism of energy. Because there was no ruptured globes in this experiment, it would seem reasonable to conclude that the fracture threshold is less than the disruption threshold of the sclera.	Given the various differences between goat and human physiologiy/anatomy, the findings need to be confirmed in human studies.

**Johnson D et al**	2016	Efficacy of Intravenous Mannitol in the Management of Orbital Compartment Syndrome: A Nonhuman Primate Model	4 / 8	Animal (Non-human primates (Macaca fascicularis monkeys))	Orbital compartment syndrome with orbital compartment pressure artificially elevated to 80mmHg.	To report the efficacy of intravenous mannitol in the treatment of orbital compartment syndrome.	A Stryker Intra-Compartmental Pressure Monitor system was used, as described by Kratky et al.	Change following intravenous infusion:<br>- mannitol: -34mmHg (mean)<br>- saline: -9.3mmHg (mean)<br><br>Further change following lateral canthotomy, inferior and superior cantholysis:<br>- mannitol and saline: 26-27mmHg (mean)<br><br>At the end of the study protocol:<br>- mannitol and saline: approximately 20mmHg (mean)	None	Over a 20-minute period between when the mannitol/saline infusion started and just before lateral canthotomy, orbital compartment and intraocular pressures decreased by a mean of 34.0 and 34.8mmHg, respectively. This rapid decrease in pressure is consistent with the use of mannitol in treating cerebral edema, where intracranial pressure is reduced in as little as 10 to 15 minutes.	While the definitive treatment of orbital compartment syndrome is lateral canthotomy and cantholysis, mannitol results in a rapid and clinically meaningful drop in orbital compartment and intraocular pressures. These findings support the routine use of mannitol in orbital compartment syndrome, especially when there is a delay in timely surgical management. This study also found a close correlation between orbital compartment and intraocular pressures during the experimental protocol. This supports the recommendations that intraocular pressure can be used as a surrogate for rbital pressure in the setting of orbital compartment syndrome.	This custom device may be used for measuring orbital compartment pressure in vivo in different settings. Feasibility needs to be further tested.
**Kratky V et al**	1990	Orbital compartment syndrome. Direct measurement of orbital tissue pressure: 1. Technique	11 / 14	Human	Normal orbits, Graves orbitopathy, other orbital disease. Before and during orbital surgery (enucleation, exenteration or decompression).	To describe the experience with the "slit-catheter" technique for direct tissue pressure measurement as applied to the orbit.	An orbital pressure probe consisting of a sterile indwelling slit catheter, a pressure transducer and a non-sterile solid-state monitor (Stryker Surgical, Kalamazoo, Michigan, USA) was used. The needle and the catheter were advanced transcutaneously into the orbit for 1.5 to 2cm, whereas the starting point was two-thirds the distance laterally along the orbital rim. The needle was then removed, while the catheter and tubing were left in place and secured with tape.	Preoperative:<br>- normal orbits (5 patients, 5 orbits): 3-6mmHg (range)<br>- Graves orbitopathy (4 patients, 7 orbits): 7-15mmHg (range)<br>- other orbital disease (2 patients, 2 orbits): 3-5mmHg (range)	None	This study establishes a normal range of orbital compartment pressure. Since normal subcutaneous tissue pressure levels are subatmospheric, these findings support the concept that the orbit functions as an enclosed compartment.	The results suggest that the orbital pressure probe may have many exciting possibilities as a clinical research tool in orbital disease, and its use will result in better understanding of orbital compartment syndromes.	This custom device may be used for measuring orbital compartment pressure in vivo in different settings. Feasibility needs to be further tested.
**McCord CD et al**	1985	Pressure-volume orbital measurement comparing decompression approaches	2 / 4	Cadaver	Simulated Thyroid Eye Disease	To measure space and orbital compartment pressure lowering that can be made available by four different orbital decompression techniques in simulated thyroid eye disease.	Two thin lateral balloons/bladders fashioned from condoms to measure pressure and volume changes in the orbit were used. Both balloons were connected to a tube for infusion of increasing volumes of water and to a Honeywell medical pressure transducer. The two pressure transducers were connected to a medical physiograph to give digital pressure readouts in mmHg. Pressure was measured during water infusion and during surgical decompression.	Mean differences following surgical decompression (lateral decompression, antra-ethmoidal decompression, 3-Wall-decompression, 4-Wall-decompressio):<br>- anterior balloon: 17.4-67.3mmHg (range)<br>- apical balloon: 21.5-80.5mmHg (range)	None	Lateral Wall decompression is inferior to the other decompression types in terms of pressure reduction.	Lateral wall decompression alone may not provide sufficient pressure reduction.	Further research is warranted to gain a better understanding of the pressure reducing effects of different orbital decompression approaches.
**Oester AE et al**	2012	Inferior orbital septum release compared with lateral canthotomy and cantholysis in the management of orbital compartment syndrome	5 / 10	Cadaver	Simulated orbital compartment syndrome	To compare the efficacy of inferior orbital septum release with lateral canthotomy and inferior cantholysis using a cadaveric model for orbital	Simultaneous orbital compartment pressures were obtained with a Synthes Compartment Pressure Monitor. The probe for the monitor was introduced into the superior lateral orbit through a large bore needle to a depth of two centimeters. The needle was then removed leaving the probe within the orbital compartment. The monitor was	Before orbital decompression: 97-123mmHg (range)<br><br>After decompression (canthotomy, cantholysis and inferior inferior septotomy): 21-47mmHg (range)	None	Cantholysis/Cantothomy and Inferior Septotomy both decrease orbital compartment pressure significantly and if sequentially performed the effect is additive, independent from the order in which they are performed.	Both procedures may be used isolated or in combination for managing orbital compartment syndrome. Since orbital compartment and intraocular pressure values correlated, intraocular pressure may be	The potential of the measuring device for assessing orbital compartment prssure in clinical settings in vivo needs to be further explored.

						compartment syndrome.	connected to the probe and provided a digital read-out of orbital pressures in mmHg.				considered a valid surrogate for orbital compartment pressure.	
**Otto AJ et al**	1996	Retrobulbar pressures measured during surgical decompression of the orbit	9 /10	Human	Thyroid Eye Disease	To investigate the pressure levels involved during orbital decompression in thyroid eye disease.	Direct orbital compartment pressure measurements were performed by placing a micro pressure transducer catheter (4 F Honeywell MTCR) in the orbit, behind the globe, and connection to a Princeton Applied Research (PAR) amplifier with a chart recorder. The transducer was inserted retrobulbarly through a trocar needle, and was sutured to the skin after withdrawal of the trocar. To investigate the force/pressure relation during surgery the force exerted upon intraorbital tissues by the surgical spatula was measured. The spatula was mounted with strain gauges (stretch sensitive elements) on both sides for registration of the exerted force.	Before orbital decompression:<br>- with compressive optic neuropathy: 28.7mmHg (mean), 17-40mmHg (range)<br>- without compressive optic neuropathy: 8.8 and 11.0mmHg<br><br>Decrease following orbital decompression:<br>- with compressive optic neuropathy: -10mmHg<br>- without compressive optic neuropathy: no change	None	Thyroid Eye Disease is associated with significantly increased orbital compartment pressure. It may be concluded from the limited results that Thyroid Eye Disease with optic neuropathy is associated with much higher pressures than without optic neuropathy. Moreover, it is evident that a decrease can already be established at the end of the decompression surgery.	The force of 250 to 300 g against the intraorbital tissues exerted intraoperatively by a normally handled spatula resulted in transient orbital compartment pressure levels of over 70mm Hg. At this pressure, dangerous impairment of the arterial vascularisation of the optic nerve and retina might ensue, especially in cases with preexisting vascular disorders. Caution needs to be exercised when maneurvering intraoperatively.<br>It appears unlikely that a direct or diffuse pressure rise will be the only cause of impairment of the nerve function. Thus, secondary incarceration might also be involved. When orbital compartment pressure has reached higher levels than the intracranial pressure, optic nerve encroachment may occur near the optic foramen, caused by orbital tissues bulging backward inside their lining periosteal membranes.	Future studies using less invasive techniques to measure orbital compartment pressure may allow an improvement in the timing of decompression surgery to prevent optic neuropathy in these patients. Cadaver studies should be performed to test this hypothesis.<br>The potential of the device used for measuring orbital compartment pressure for clinical practice needs to be further tested.
**Ramesh S et al**	2018	Medial Buttressing in Orbital Blowout Fractures	5 / 10	Cadaver	Simulated orbital blowout fracture following trauma simulation	To study whether ethmoidectomy predisposes the orbit to medial wall fracture with lesser trauma.	Same as Rieman et al	During orbital impact: 2-43.7mmHg (range)	None	This study showed that endoscopic ethmoidectomy in fresh cadavers reduces impact energy necessary to induce orbital fracture and increases the prevalence of medial wall involvement.	Clinicians may wish to counsel patients undergoing endoscopic sinus surgery about these relative risks.	The potential of this measuring device for assessing orbital compartment pressure in clinical settings in vivo needs to be further explored.
**Reedy BK et al**	1999	The Direct Effect of Intraorbital Pressure on Orbital Growth in the Anophthalmic Piglet	16 / 16	Animal (Yorkshire Piglets)	Controls (Enucleation only, 1), Enucleation and orbital expansion at 20mmHg, 2), Enucleation and orbital expansion, 3)	To simulate anophthalmos and to study the effect of different orbital compartment pressures on orbital growth and thus to gain a better understanding of the management of post-enuclation (e.g. due to retinoblastoma) conditions in children.	Spherical tissue expanders were utilized as the orbital expanders with a separate injection port. Pressure was monitored by an electronic manometer that was calibrated daily (Universal Biometer, DPM III; BioTeck, Lafayette, Ind.).	In controls (n=6): 17mmHg (mean)<br><br>Experimental groups 2 (enucleation and orbital expansion, n=5): artificially maintained at 20mmHg<br><br>Experimental group 3 (enucleation and orbital expansion, n=5): artificially maintained at 60mmHg	None	There was a direct relationship between orbital compartment pressure and growth of the bony orbit in the radial dimension.	Orbital compartment pressure at normal physiologic pressure can stimulate normal orbital growth in the neonatal facial skeleton. Above-normal orbital compartment pressure can accelerate orbital growth.	The optimal pressure in a child's orbit needs to be determined.

**Rieman CD et al**	1999	Direct orbital manometry in healthy patients	18 / 18	Human	Healthy orbits	To determine orbital compartment pressure and orbital tissue compliance in normal patients.	The Atkinson 23-gauge blunt-tipped retrobulbar needle (Storz Inc., St. Louis, MO) used for the anesthetic injection served as a pressure probe. The needle was placed into the intraconal-retrobulbar space. The pressure in the retrobulbar needle was measured by an arterial/venous pressure transducer kit commonly used for measuring hemodynamic parameters (Mean Kit, list no. 46067-19, Abbot Critical Care Systems, Abbott Laboratories, North Chicago, IL). The pressure transducer was attached to the Mono Eagle Marquette Series 2 patient monitoring system (Marquette Electronics, Milwaukee, WI), which yielded both alphanumeric and graphic representations of the pressure. A syringe infusion pump (Model A540A, Baxter Diagnostics Inc., Deerfield, IL) was used to deliver a 10-ml/min flow of anesthetic solution through the entire system and out through the needle tip to maintain a patent fluid column from the pressure transducer to the orbital compartment and to prevent measurement artifact from orbital fat occluding the needle tip. This infusion rate was low enough to prevent interference with accurate pressure measurements. A stepwise injection of 5 ml of local anesthetic was delivered in three 1.66-ml increments.	At Baseline: 4.0±1.5mmHg (mean±SD),<br>After 1.66ml injection: 6.3±1.7mmHg (mean±SD)<br>After 3.33ml injection: 8.8±2.1mmHg (mean±SD)<br>After 5ml injection: 11.6±2.6mmHg (mean±SD)<br><br>Five minutes after injection: 6.6±1.9mmHg (mean±SD)	None	The authors' orbital manometer safely determined orbital compartment pressure in normal orbits. Retrobulbar anaesthetic injection causes consistent measurable changes in orbital compartment pressure.	Directly assessing orbital dynamics in vivo may prove useful both as an adjunct in the clinical evaluation of patients with disorders resulting in an orbital compartment syndrome as well as in assessing the risk of retrobulbar injection in orbits at greater risk for complications from this procedure.	The device may be helpful for assessing the progression of different orbital diseases. Further research is necessary to explore the potential of the device in this respect.
**Rieman CD et al**	1999	Direct orbital manometry in patients with thyroid-associated orbitopathy	53 / 53	Human	Thyroid Eye Disease; healthy orbits	To determine orbital compartment pressure and orbital compartment compliance in patients with and without thyroid eye disease.	Same as above.	Baseline:<br>- controls (n=35): 4.4±2.2mmHg (mean±SD)<br>- Thyroid Eye Disease (n=18): 9.7±4.8mmHg (mean±SD)<br><br>Immediately following injection of 5ml anesthetic:<br>- controls: 12.0±3.6 (mean±SD)<br>- thyroid eye disease: 20.1±5.8 (mean±SD)<br><br>5 minutes after injection of 5ml anesthetic:<br>- controls: 6.7±2.2mmHg (mean±SD)<br>- thyroid eye disease: 13.7±4.1mmHg (mean±SD)	None	The described procedure is a safe minimally invasive method to directly measure orbital compartment pressure. These data support the notion of an orbital compartment syndrome possibly contributing to compressive optic neuropathy in addition to, or even instead of, direct optic nerve compression by enlarged extraocular muscles in some patients with thyroid eye disease. In our control patients, we were able to demonstrate a statistically insignificant trend of older patients having more lax orbits.	Orbital manometry might prove useful during the performance of retrobulbar injections on patients at risk for complications from this procedure. Direct measuerement of orbital compartment pressure may prove useful in clinically assessing the progression of orbital disease and thus in optimizing the timing of medical or surgical interventions.	The effect of general anesthesia on orbital dynamics is not adequately addressed by the present data and remains to be clarified. Further study of baseline orbital comparment pressure and retrobulbar injection–associated increases in orbital compartment pressure may also be useful in explaining the phenomenon of posterior pressure observed during intraocular surgery. More data that specifically correlate orbital dynamics to clinical presentation and progression of Thyroid Eye Disease are needed to validate this conjecture, that lower orbital compliance is a

												risk factor for developing compressive optic neuropathy in patients with Thyroid Eye Disease.
**Stanley RJ et al**	1989	Space-occupying orbital lesions: can critical increases in intraorbital pressure be predicted clinically?	9 / 13	Cadaver	Simulated intraorbital space-occupying lesions	To determine whether critical intraorbital pressure changes could be predicted by noninvasive clinical measurements.	A Fogarty occlusion catheter (American Edwards Laboratories, Santa Ana, CA) was used. The catheter had a 22 Fr maximum size over the deflated balloon. The catheter was inserted into the intraconal space of the orbit through the optic canal. Pressures were measured during intraorbital volume addition with a Statham P 23 dB pressure transducer and recorded on a Beckman R 611 recording oscillograph.	At baseline: close to 0mmHg<br><br>Following 12ml intraorbital volume addition: up to 600mmHg and higher.	None	The results of these studies suggest that indirect clinical measurements of globe proptosis and excursion produced by standard forces permit an estimate of intraorbital compartment pressure	Because orbital compartment pressure is one of the factors that may lead to vision loss in intraorbital space-occupying lesions, these measurements may prove to be clinically useful.	Further research to develop techniques for minimally invasive direct measurement of orbital compartment pressure may be warranted.
**Strand AT et al**	2017	Canthal cutdown for emergent treatment of orbital compartment syndrome	5 / 10	Cadaver	Simulated orbital compartment syndrome	To evaluate the use of a “canthal cutdown” technique in orbital compartment syndrome in a cadaveric model.	Orbital compartment pressure was monitored via two compartment monitoring systems. A Stryker Intracompartmental Pressure Monitor System (Stryker Instruments, Kalamazoo, MI) was placed at he lateral canthal angle into the orbit directed toward the superior temporal quadrant of the orbit. The probe was advanced 40mm of its 60mm length into the orbit. A Compass Compartment Pressure Monitor (Mirador Biomedical, Inc., Seattle, WA) attached to a 40-mm, 18-gauge needle was passed medially through the caruncle and directed posterior. The probe was advanced 30 mm into the orbit. The exact location of the probe tips was not radiologically identified.	At baseline:<br>- Stryker; 7mmHg (mean), 4.89-9.10mmHg (range)<br>- Compass: 5mmHg (mean), 3.88-6.92mmHg<br><br>Following 20ml intraorbital volume addition:<br>- Stryker: 115mmHg (mean), 86.27-143.50mmHg (range)<br>- Compass: 89mmHg (mean), 59.38-118.6mmHg (range)<br><br>Post cutdown:<br>- Stryker: 20mmHg (mean), 9.28-31.31 (range)<br>- Compass: 17mmHg (mean), 11.12-22.28mmHg (range)	None	In summary, the canthal cutdown technique appears to provide greater orbital compartment pressure reductions than does canthotomy and cantholysis alone.	The septal defect and orbital septae interruptions created by the cutdown technique may be beneficial in cases of both static and dynamic orbital compartment pressure where there is insufficient clinical improvement after standard canthotomy and cantholysis.	Measurements with the Stryker and the Compass Compartment Pressure Monitor were in line, advocating reliability. The potential for clinical use is yet to be further evaluated.
**Zhou H et al**	2007	Direct orbital manometry in normal and fractured orbits of Chinese patients	40 / 80	Human	Unilateral orbital wall fractures undergoing surgical repair, Contralateral eyes as intraindividual controls	To directly measure orbital compartment pressure in normal and fractured orbits of Chinese patients before and after orbital fracture repair. To investigate the changes in orbital compartment pressure and intraocular pressure after surgery and to evaluate any correlation between them.	A direct orbitonometer, comprised mainly of a retrobulbar catheter, pressure transducer, and pressure monitor, was designed and assembled. An 18-G intravenous catheter was connected to a pressure transducer through a pressure monitoring. The pressure transducer was connected to a pressure monitor to record measurements both graphically and numerically. With the patient in upgaze, the catheter with needle inside was inserted through the lateral one third of the lower lid and advanced 3.5 cm into the retrobulbar space between the inferior rectus and lateral rectus muscles. The needle was then drawn back, leaving the catheter in place. The height of the pressure transducer was adjusted to the same height as the catheter opening, and then the pressure monitor recorded the DOT value both graphically and numerically. The very slow velocity of continuous infusion (0.1 mL/hr) by the syringe infusion pump ensured that any added liquid would not raise the orbital compartment pressure artificially. Orbital	control side: 3-7mmHg (range)<br><br>affected side: 3-10mmHg (range)	None	Direct orbital manometry allows for reliable measurement of orbital compartment pressure and is useful for early detection of increasing orbital tension during or after orbital surgery. Both orbital compartment pressure and intraocular pressure were significantly decreased after orbital fracture and increased after orbital fracture repair; furthermore, orbital compartment pressure was well-correlated with intraocular pressure.	Both orbital compartment and intraocular pressure increased significantly in the affected orbits immediately after orbital repair and reconstructive surgery. Orbital compartment pressure was positively correlated with intraocular pressure. This positive correlation may mean that orbital compartment pressure can be readily monitored simply by measuring intraocular pressure during or after fracture surgery.	The potential of this custom device for measuring orbital compartment pressure is yet to be fully explored.

							pressure was measured before and immediately after surgery in each patient.					
**Zoumalan CI et al**	2008	Evaluation of intraocular and orbital pressure in the management of orbital hemorrhage: an experimental model	5 / 10	Cadaver	Simulated orbital hemorrhage	To evaluate orbital compartment pressure, intraocular pressure, and the effectiveness of canthotomy, cantholysis, and septolysis using an experimental orbital hemorrhage model.	A Stryker Intracompartmental Pressure Monitor System (Stryker Instruments, Kalamazoo, Michigan) was used for measuring orbital pressure. The Stryker Intracompartmental Pressure Monitor System has an indwelling catheter system that was inserted adjacent to the 10-mL syringe of whole blood used for injecting the volume into the orbit to simulate elevated orbital compartment pressure. This way, the orbital compartment pressure could be measured continuously.	At baseline: 4.1±2.7mmHg (mean±SD)<br><br>After injecting 22 mL of whole blood: 68.4±32.2 (mean±SD)<br><br>With additional injection of blood, orbital compartment and intraocular pressures increased rapidly.<br>After 10 mL: from 20.4±10.1mmHg to 65.7± 28.4mmHg (mean±SD)<br><br>After canthotomy: from 68.4±32.2 to 36.5±18.3mmHg (mean±SD)<br><br>After cantholysis: from 36.5±18.3 to 24.8±10.1mmHg (mean±SD).<br><br>After septolysis: further reduction to 20.4±10.1mmHg (mean±SD)<br><br>Total reduction following all three procedures: 48.0±27.2mmHg (mean±SD) (-70%)<br><br>Parallel changes in intraocular pressure were observed, showing a total decrease of 50.0±18.1mmHg (mean±SD). (-58.7%)	None	There was a very close correlation between intraocular pressure and orbital compartment pressure (r=0.97), with mean intraocular pressure remaining an average of 11 mmHg greater than orbital compartment pressure. This study substantiates the effectiveness of canthotomy and cantholysis in human cadavers using human blood.	Intraocular pressure may be used clinically as a surrogate for orbital compartment pressure. However, clinicians should be aware that some variability exists and not rely entirely on intraocular pressure as an indicator of elevated orbital compartment pressure. Canthotomy, cantholysis and septolysis are efficient in lowering elevated orbital compartment pressure.	Evaluating the extension of an orbital hemorrhage to adjacent tissue and/or cavities is a potential area for future investigation that might prove interesting. Comparison with a control orbit in which canthotomy, cantholysis, and septolysis are not performed might also provide useful information regarding the natural history of an orbital hemorrhage. The potential of the Stryker pressure monitoring device for measuring orbital compartment pressure in clinical settings remains to be fully explored.
